# Managing and Controlling Stress Using mHealth: Systematic Search in App Stores

**DOI:** 10.2196/mhealth.8866

**Published:** 2018-05-09

**Authors:** David Blázquez Martín, Isabel De La Torre, Begonya Garcia-Zapirain, Miguel Lopez-Coronado, Joel Rodrigues

**Affiliations:** ^1^ Department of Signal Theory and Communications, and Telematics Engineering University of Valladolid Valladolid Spain; ^2^ Faculty of Engineering University of Deusto Bilbao Spain; ^3^ Instituto de Telecomunicações Portugal Portugal

**Keywords:** apps, control, management, mHealth, stress

## Abstract

**Background:**

Traditional stress management techniques have been proven insufficient to tackle the needs of today’s population. Computational-based techniques and now mobile health (mHealth) apps are showing promise to enable ease of use and access while educating end users on self-management.

**Objective:**

The main aim of this paper was to put forward a systematic review of mHealth apps for stress management.

**Methods:**

The scenario chosen for this study consists of a sample of the most relevant mHealth apps found on the British and Spanish online stores of the two main mobile operating systems: iOS and Android. The apps have been categorized and scored base on their impact, presence, number of results, language, and operating system.

**Results:**

A total of 433 different mobile apps for stress management was analyzed. Of these apps, 21.7% (94/433) belonged to the “relaxing music” category, 10.9% (47/433) were in the “draw and paint” category, 1.2% (5/433) belonged to the “heart rate control” category, and 1.2% (5/433) fell under “integral methodology.” Only 2.0% (8/433) of the apps qualified as high or medium interest while 98.0% were low interest. Furthermore, 2.0% (8/433) of the apps were available on both iOS and Android, and 98% of apps ran on only one platform (iOS or Android).

**Conclusions:**

There are many low-value apps available at the moment, but the analysis shows that they are adding new functionalities and becoming fully integrated self-management systems with extra capabilities such as professional assistance services and online support communities.

## Introduction

Work-related mental health problems are one of the main causes of work leave within the countries of the Organization for Economic Co-operation and Development [1]. Research carried out by the European Agency for Safety and Health at Work shows that 28% of employees recognized having suffered stress in their working place; in addition, it is estimated that stress is the main factor in 50% to 60% of lost working days [2-3]. The increased pace at the workplace and in everyday life is dramatically increasing the number of stressful situations to which people are exposed [4].

Stress is strongly associated with mental health problems such as depression [2]. Preventive treatments for high-risk individuals have proven successful in reducing pathologies associated with mental health problems as a consequence of high stress levels [5-6]. Stress management therapy programs are usually focused on teaching individual techniques for stress relief and generally extend several weeks or months [7-8]. A very small portion of the public health budget is allocated for medical problems associated with stress, with an even smaller amount allocated for preventive interventions [9]. New tools to facilitate welfare self-management should be accessible wherever and whenever they are needed. Stress management therapy refers to the techniques used by therapists, doctors, and psychiatrists who help stressed persons relieve their tension and stress. Some stress management therapies include interaction, biofeedback, relaxation, cognitive behavior therapy (CBT), and different exercises (yoga or meditation) [8].

Personal health technologies look promising in order to support people in health and welfare self-management. For example, several studies have gathered positive impact in the use of Web-based therapies for the treatment of health problems related to stress and anxiety [8-13].

Web-based therapies are accessible 24/7 and can be customized depending on user needs and allow the user to avoid the embarrassment of visiting a professional. The British National Health System (NHS) offers patients 2 different Web-based CBTs, but browser-based interventions aren’t as attractive now that mobile apps have become popular [14-16].

User response in terms of estimated delay is characterized by the time window data-cleaning [17-19]. Response times depend on geography, platform (Android or iOS), and other factors. Other authors study mobile device-to-device video distribution that leverages the storage and communication capacities of smartphones [19].

This research tries to study in depth the status of stress-related mobile apps and the different therapies and methodologies being used and tries to forecast the patterns that will define stress-related mobile apps in the near future. In this paper, the apps have been divided into different categories such as self-help, heart rate control, integral methodology for the management and prevention of stress, hypnosis, games, images, meditation and breathing, relaxing music, draw and paint, yoga, guided relaxation, and others. This classification has followed the criteria of the authors and the queries to users.

## Methods

**Figure 1:**
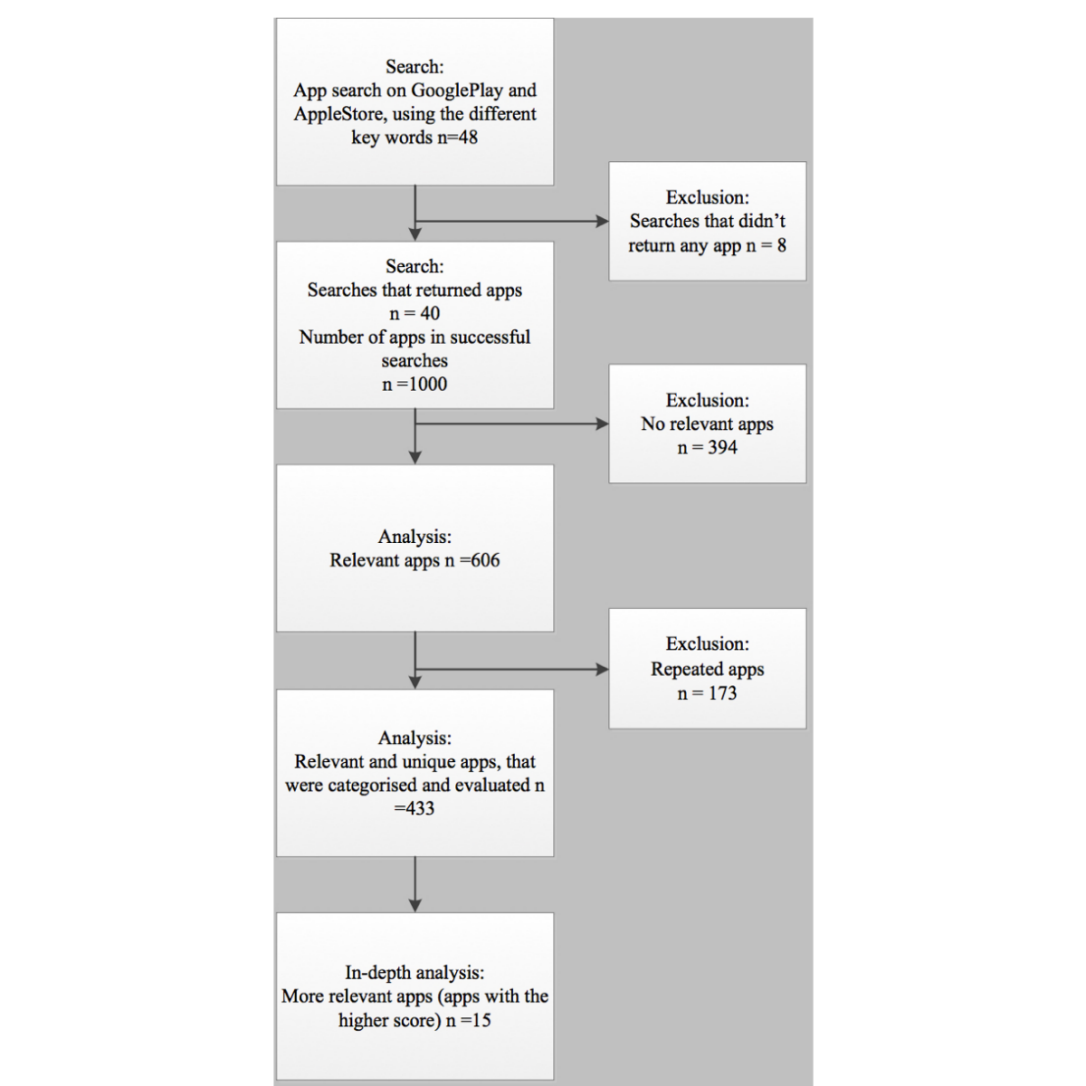
Flowchart management and control of stress-related mobile app research.

In this study, 24 keywords were chosen. On June 2017, 48 searches were carried out in the Spanish and English virtual stores of the 2 main operating systems for mobile phones, Google Play and App Store. Eight searches returned no results. The remaining 40 searches returned a total of 1000 apps, with 606 being relevant (see Figure 1) and 433 unique. Characteristics of these 433 unique apps were recorded, and the apps were categorized.

The 15 most relevant apps have been chosen from the “Most relevant” category in the app stores. If more than 5 consecutives apps were identified as not relevant (eg, games), the apps chosen for that search were limited at all the previous results excluding those 5.

The following information was recorded for each app: country, language, operating system, and free or pay per use. Additionally, after we installed and tested them, all the apps were sorted according to different categories (see Table 1).

After the apps were recorded, they were scored based on the following criteria:

- Language: 2 points were given if the app was available only in English or Spanish; 4 points if it was available in both languages.
- Operating system: 2 points were given if the app was available only in Android or iOS; 4 points if the app was available in both.
- Keywords: 1 point was given for every search where the app appeared.
- Relevancy: 10 points were given to the apps sorted as “high interest,” 7 for the apps considered “medium interest,” and 0 for the apps with “low interest.”

Some of the apps with higher scores were analyzed in depth in order to identify the methodology used in their design.

## Results

In this section, we analyze stress-related mobile apps currently available in the United Kingdom and Spain. A total of 433 different apps were installed, tested, analyzed, categorized, and scored.

As can be seen in Table 1, about one-fifth (94/433, 21.7%) of the apps belong to the “relaxing music (songs, natural sounds)” category, and these apps included either relaxing songs or sound that are typically associated with relaxation and inner peace like sea, rain, mountains, or lakes. Mandalas have become well known thanks to the media in the last several years and represent most of the 10.9% (47/433) of apps in the “draw and paint” category.

Apps about integral methodology are based on CBTs including cognitive therapy, problem-solving therapy, dialectical behavior therapy, metacognitive therapy, rational-emotive behavior therapy, cognitive processing therapy, mindfulness-based cognitive therapy, cognitive-behavioral analysis system of psychotherapy, and schema-focused therapy.

Of note, 6 apps focused on guided relaxation are also included in the 15 most relevant apps. Its 10 points of median with a standard deviation of 6.77 proved that despite having 6 high scoring apps in this group, most of the apps obtained the minimum possible score points.

When the apps were categorized and relevance was evaluated, only 1.8% (8/433) of the apps qualified as high or medium interest while 98.0% were low interest (Table 2).

**Table 1:** Mobile app categorization statistics (n=433).

Categories	Amount, n (%)	Scores				
		Max	Min	Average	Median	SD
Self-help	47 (10.9)	16	10	10.7	10	1.67
Heart rate control	5 (1.2)	22	10	13.2	10	4.66
Integral methodology for the management and prevention of stress	5 (1.2)	22	10	18.4	20	4.27
Hypnosis	6 (1.4)	16	10	12.3	11	2.68
Images	1 (0.2)	10	10	10.0	10	0
Games	69 (16.0)	20	10	10.6	10	2.08
Meditation and breathing	66 (15.2)	18	10	10.8	10	1.85
Relaxing music	94 (21.7)	28	10	11.3	10	2.50
Not relevant	47 (10.9)	16	10	10.4	10	1.23
Draw and paint	47 (10.9)	16	10	10.9	10	1.28
Guided relaxation	34 (7.9)	36	10	13.3	10	6.77
Yoga	12 (2.8)	16	10	10.7	10	1.69

**Table 2:** App interest statistics (n=433).

Interest	Amount, n (%)	Scores			
		Max	Min	Average	Median
Low	33 (10.8)	22	10	10	1.82
Medium	74 (17.0)	17	17	20	0.80
High	107 (24.6)	36	20	27	5.53

**Table 3:** App platform and language availability (n=433).

Characteristic		Amount, n (%)
**Platform**		
	Android	253 (58.4)
	iOS	172 (39.7)
	Both	8 (1.8)
**Language**		
	English	225 (52.0)
	Spanish	200 (46.2)
	Both	8 (1.8)

Only 1.8% (8/433) of the apps are available in both iOS and Android environments (Table 3). Android is the world's most popular mobile platform. According to Gartner [20], Android and iOS accounted for 99.6% of all mobile phone sales in the fourth quarter of 2016. Many apps only run on one platform (Android or iOS), and this is true with health care apps as well.

This study also focused on analyzing the country and language where the apps were found; 46% of the apps were only found in the Spanish store. Only 2% of the apps were available in both Spain and the United Kingdom, even though it is well known that most of the app developers publish their apps in more than 1 country at a time. This is probably happening due to the high number of different apps offered, where being in the top 25 relevant apps in getting more difficult.

## Discussion

### Principal Findings

App stores are overloaded with different apps that provide no added value to the end user; more people have now access to the tools and knowledge needed to develop their own apps with all their advantages and disadvantages.

Almost a third (304/1000, 30.40%) of the initial group of apps were discarded even before installing them because the descriptions and screenshots on the app store site showed that they were not stress management apps. After installation and testing of the apps, 10.9% (66/606) were categorized as not relevant and 16.0% (97/606) as games.

It has not been possible to find research that demonstrates that certain type of games would improve the management and prevention of stress. Therefore, it can be concluded that from the initial 1000 apps, 51.00% (51/1000) of the apps do not pertain to the management and prevention of stress even though they were listed as the 15 most relevant apps for the search keywords [10-12].

Focusing on the categorization results, it can be seen that 1 out of every 4 apps was categorized as “relaxing music (songs, natural sounds).” This is likely linked to the traditional believe that relaxing sounds and music can eventually help with stress relief. Studies have proven this point but failed to show improvement on long-term stress management and prevention [10-13]. Similar justification may be why 15.2% of apps are based on breathing and relaxation techniques, which have also been associated with improvement on anxiety and stressful situations.

Several studies have shown the benefits of these techniques and methodologies over stressful situations but have yet been able to prove positive impact on the management and prevention of stress in the long term [21-24].

### Limitations

Some of the limitations of this review are the chosen languages of the apps (just Spanish and English), the keywords for the search are limited, and authors cannot guarantee that all the relevant apps have been retrieved.

### Conclusions

The methodology applied in this study has simplified the identification of clear patterns that show the trend being followed for the development of mobile apps for the management and prevention of the stress.

Several apps offer very limited exercises or techniques, but these exercises have shown improvement for stress relief. Apps like “Qi Gong Meditation” [25] and “Relax lite” [26] offer activities from yoga exercises, breathing and meditation exercise to painting mandalas and listening to relaxing sound or self-aids.

Apps like Pacifica [27], Calm [28], or SAM [29] based their full program on acceptance and commitment techniques and try to introduce the user to a self-learning introspection process while at the same time offering tools for the self-management of the stress. In additional to offering several different exercises similar to the other group, these apps offer tools to enable the user to track day-to-day activities (sleep time, eating, drinking, or sun time) and dreams and fears. They also offer tools to share and speak about previous stressful situations or evaluate mood rate. They often include online communities for experience sharing.

Professional assistance services provided by some apps like SAM and iDstress [30] serve as an added value and a step forward in the integration of mobile apps and acceptance and commitment therapies.

There is no research that shows the real influence of these apps for the reduction and management of stress, but it is worth mentioning the revolution regarding easy access and learning of techniques and methodologies for stress management. It is expected that the mobile apps related to management and control of stress will evolve in the coming years adding new functionalities until they become fully integrated self-management systems, and more and more apps are likely to include the above mentioned professional assistance services and online support communities.

## Abbreviations

CBT: cognitive behavioral therapy
mHealth: mobile health
NHS: National Health System
